# Quality of Life of Patients With Osteosarcoma in the European American Osteosarcoma Study-1 (EURAMOS-1): Development and Implementation of a Questionnaire Substudy

**DOI:** 10.2196/14406

**Published:** 2019-09-26

**Authors:** Gabriele Calaminus, Meriel Jenney, Lars Hjorth, Katja Baust, Mark Bernstein, Stefan Bielack, Patricia De Vos, Pancras C W Hogendoorn, Gordana Jovic, Mark Krailo, Kiana Kreitz, Neyssa Marina, Babasola O Popoola, Cristina Sauerland, Sigbjørn Smeland, Carmen Teske, Clara V Schweinitz, Jeremy Whelan, Andreas Wiener, Matthew R Sydes, Rajaram Nagarajan

**Affiliations:** 1 Department of Pediatric Hematology and Oncology University Hospital Bonn Bonn Germany; 2 Children & Women's Services Clinical Board University Hospital of Wales Cardiff United Kingdom; 3 Pediatrics, Department of Clinical Sciences Lund Skane University Hospital Lund University Lund Sweden; 4 IWK Health Centre Dalhousie University Halifax, NS Canada; 5 Cooperative Osteosarcoma Study Group Klinikum Stuttgart - Olgahospital Stuttgart Germany; 6 Department of Pediatric Hematology and Oncology Gent University Hospital Gent Belgium; 7 Department of Pathology Leiden University Medical Center Leiden Netherlands; 8 MRC Clinical Trials Unit at UCL Institute of Clinical Trials and Methodology University College London London United Kingdom; 9 Department of Preventive Medicine Keck Medical Center at the University of Southern California Los Angeles, CA United States; 10 Children's Oncolgy Group Arcadia, CA United States; 11 Institute of Biostatistics and Clinical Research University of Muenster Muenster Germany; 12 Five Prime Therapeutics Inc South San Francisco, CA United States; 13 Division of Cancer Medicine and Scandinavian Sarcoma Group Oslo University Hospital Oslo Norway; 14 Institute for Clinical Medicine University of Oslo Oslo Norway; 15 Department of Oncology University College Hospital London United Kingdom; 16 West German Proton Therapy Centre Essen Essen Germany; 17 Department of Pediatrics University of Cincinnati College of Medicine Cincinnati, OH United States; 18 Division of Oncology Cincinnati Children's Hospital Medical Center Cincinnati, OH United States

**Keywords:** osteosarcoma, quality of life, cancer, child, adolescent, young adult, observational study, sarcoma, survivors of childhood cancer

## Abstract

**Background:**

The quality of life (QoL) of patients with osteosarcoma (OS) may be adversely affected by the disease or its treatment. Therefore, it is important to understand the QoL of patients undergoing treatment for OS to improve the QoL. We report on the first prospective international QoL study that was embedded within a large randomized clinical trial from 4 national study groups.

**Objective:**

This paper aimed to describe the QoL study development, methodology, accrual details, and characteristics of the QoL cohort.

**Methods:**

A total of 2260 patients registered in the EURopean AMerican Osteosarcoma Study-1 (EURAMOS-1), of whom 97.92% (2213/2260) were eligible for the optional QoL assessment and could participate in terms of questionnaire availability. Overall, 61.86% (1369/2213) of patients and/or proxies completed the QoL evaluation at the first assessment time point (E1) after the start of preoperative treatment. The QoL measures used (self- and/or proxy reports) depending on the patient’s age and national study group. Participants and nonparticipants in the ancillary QoL study were compared regarding relevant demographic and disease-related characteristics at registration in the trial.

**Results:**

The participation rate at time point E1 did not differ with regard to age, gender, the occurrence of pathological fracture, or the presence of any metastases at diagnosis. No differences were found regarding the primary tumor site. Only the national study group affiliation had an influence on participation. Participation decreased linearly with trial progress up to 20% at the final time point of QoL assessment.

**Conclusions:**

This study demonstrates the feasibility of international cooperation for the purpose of assessing and understanding the QoL of pediatric and adolescent/young adult patients with cancer. Future outcomes of this QoL substudy will help to adapt interventions to improve QoL.

## Introduction

Treatment outcomes for patients diagnosed during adolescence and young adulthood with the most common bone sarcomas, osteosarcoma (OS), and Ewing sarcoma have improved over the past 30 years with the evidence-based introduction of intensive chemotherapy, wide-margin surgery, and, for some, radiation treatment [1-5]. The 5-year survival rate has improved especially for patients aged younger than 25 years [6]. Bone sarcomas and their treatments have a direct impact on organ function, activities of daily life, mobility, and quality of life (QoL), including emotional and physical well-being [5,7,8]. The impact on QoL is a further concern as the majority of patients are diagnosed during adolescence and young adulthood, a crucial time for achieving developmental milestones. As expected, children, adolescents, and young adults diagnosed with bone sarcomas generally report lower levels of health-related quality of life (HRQoL) after surgery compared with the general population, within the domains of physical functioning and overall well-being [8-10]. In addition to physical functioning, patients receiving treatment for a high-grade bone sarcoma also show significantly poorer social functioning [8,10]. This includes lower levels of autonomy and independence when compared with matched healthy peers [8]. The intensive treatment regime as for high-grade bone sarcoma can also compromise QoL [11]. To date, the majority of studies have lacked large sample sizes and standardized treatment and have utilized varying QoL measures [12]. Furthermore, most former studies reported QoL only after surgery; only a few studies conducted prospective assessments from diagnosis to completion of therapy [13], and no study so far has been reported in the setting of a randomized trial.

Describing the impact of therapy on QoL from the patients’ perspective will lead to a better understanding of the short- and long-term treatment-related side effects and how they can best be managed to improve patient-centered care [12]. In addition, improving QoL during and after bone sarcoma treatment is thought to improve satisfaction and compliance with care and clinician-patient/family communication, which subsequently improves treatment decision [14,15]. Furthermore, through early QoL assessment, undiagnosed psychosocial and physical morbidities can be assessed [15] and potential interventions can be implemented early during treatment. The objective of this study was to assess QoL during and after OS therapy in the context of the EURopean AMerican Osteosarcoma Study-1 (EURAMOS-1). In this paper, we describe the prospective design of the EURAMOS-1 QoL assessment at 4 timepoints, the initial characteristics and participation rates of the study cohort at registration. In addition, we have explained the QoL substudy processes in detail.

## Methods

### Brief Characteristics and Inclusion Criteria of the European American Osteosarcoma Study-1 Trial

**Figure 1:**
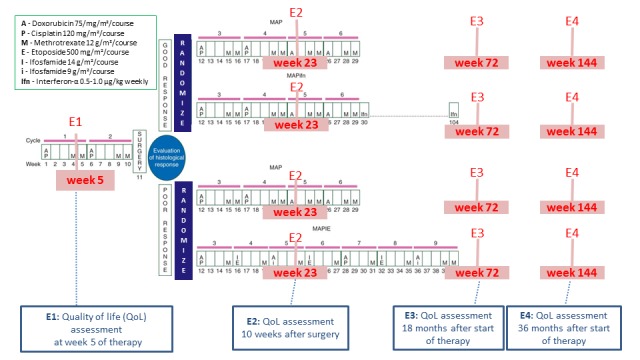
Timepoints for QoL assessment during treatment in EURopean AMerican Osteosarcoma Study-1 (EURAMOS-1). E1: first assessment timepoint; E2: second assessment timepoint; E3: third assessment timepoint; E4: fourth assessment timepoint; QoL: quality of life.

EURAMOS-1 contained 2 randomizations (4 treatment arms) to test treatment strategies for resectable high-grade skeletal OS based on histological response to preoperative chemotherapy (ISRCTN 67613327). The full details are presented elsewhere [16-19]; for an overview on the trial design, see Figure 1. The study recruited patients between 2005 and 2011. Overall, 17 countries from 4 study groups participated in the trial. The participating study groups were the Children’s Oncology Group (COG), the Cooperative Osteosarcoma Study (COSS) group, the European Osteosarcoma Intergroup (EOI), and the Scandinavian Sarcoma Group (SSG). All participating countries are listed by study group in Multimedia Appendix 1. QoL was assessed prospectively as a secondary outcome measure in all 4 treatment arms across 4 timepoints during and after treatment.

### Inclusion Criteria for Quality-of-Life Assessment Within European American Osteosarcoma Study-1

Patients were eligible for inclusion if the following criteria were fulfilled: diagnosis of previously untreated resectable high-grade OS (any site except craniofacial sites) and they were diagnosed between the ages of 5 years and 40 years.

### Questionnaires

No single questionnaire was appropriate for use in all countries for all ages at the time of trial planning. Therefore, the investigators compromised on the use of 1 questionnaire for adults (≥16 years; European Organization for Research and Treatment of Cancer—Quality of Life-Core Questionnaire C30 [EORTC-QLQ-C30]) [20] and 2 age-adapted questionnaires for pediatric patients (self-report and parallelized proxy report): Pediatric Quality of Life Questionnaire (PEDQoL) [21,22] in Central Europe and Scandinavia and Pediatric Quality of Life Inventory (PedsQL) [23,24] in North America and EOI-related countries (eg, United Kingdom or Belgium). QoL assessment was not possible in Hungary, Finland, and the Czech Republic because of a lack of validated translations of the QoL measures as of the time of study development. Wherever possible, the patient completed his/her own questionnaire and a parent filled in a (additional) proxy questionnaire until the patient turned 18 years.

#### European Organization for Research and Treatment of Cancer—Quality of Life-Core Questionnaire C30

The EORTC-QLQ-C30 is a patient-reported questionnaire that has 8 domains assessing particular aspects significant to adult patients with cancer: 5 functional domains (*Physical*=PF, *Role*=RF, *Cognitive*=CF, *Emotional*=EF, and *Social Function*=SF) and 3 symptom scales (*Fatigue*=FA, *Pain*=PA, and *Nausea and Vomiting*=NV). In addition to these scales, there is a global QoL scale and several single items assessing often-reported symptoms (dyspnoe, insomnia*,* appetite loss, constipation, diarrhea, and financial difficulties). Psychometric properties of the EORTC-QLQ-C30 are proven; the questionnaire is validated cross-culturally in different languages and used in prospective clinical trials in adult patients [25,26]. The EORTC-CLC-Q30 data were divided into 2 age groups (16-17 years and ≥18 years). This allowed us to compare a group that mostly will be treated within a pediatric setting and a group of adult participants.

#### Pediatric Quality of Life Inventory

The PedsQL is a modular questionnaire instrument designed to measure HRQoL in children and adolescents aged between 2 and 18 years. The 23-item PedsQL 4.0 Generic Core Scale implemented in this study assesses the domains *physical functioning* (8 items), *Emotional Functioning* (5 items), *Social Functioning* (5 items), and *School Functioning* (5 items). In addition, a Psychosocial Health Summary Score can be derived from the questionnaire [23]. A 5-point response scale is utilized across child self-reports for ages 8 to 18 years and parent proxy reports (*0=never a problem* to *4=almost always a problem*). The aggregated reference data of international cohorts (eg, the United States and Great Britain) are available according to the age groups expected in this study [23,27,28].

#### Pediatric Quality of Life Questionnaire

This cancer-specific questionnaire was developed to assess QoL in children [21,22]. It contains 48 items in which 6 domains can be identified: *Physical Functioning and Pain* (9 questions), *Emotional Functioning* (6 questions), *Body Image* (9 questions), *Social Functioning—Friends and Family* (12 questions), *Cognition* (6 questions), and *Autonomy* (6 questions), as well as 2 questions about general well-being. Reference data (raw data as well as aggregated data) from unselected German healthy controls are provided according to the age groups expected in this study [22].

### Assessment Timepoints

QoL was measured prospectively at 4 timepoints reflecting important therapy milestones. The initial assessment at timepoint 1 (E1) was planned at week 5 after the start of preoperative chemotherapy (±1 week) but before surgical resection. E1 was debated considerably as investigators were interested in pretreatment QoL assessments, but it was recognized that it is difficult to obtain these data before initiation of treatment, and this would result in missing QoL forms for that timepoint. Timepoint 2 (E2) was planned 10 weeks after definitive surgery for a primary tumor (± 2 weeks) as a short-term assessment following surgery. Timepoints 3 (E3) and 4 (E4) were planned 18 and 36 months after the start of therapy (± 1 month), respectively, and were in place to assess long-term outcomes following therapy. An additional timepoint between E2 and E3 was considered, but it was felt to be too burdensome. This prospective assessment across the different treatment arms allows for cross-sectional comparisons (Figure 1) and for changes across time. To include as much information as possible, a few delayed questionnaires were also taken into account for analysis if the questionnaire was received in a comparable treatment period (eg, if timepoint E1 was completed before surgery).

### Design, Organization, and Study Structure

**Figure 2:**
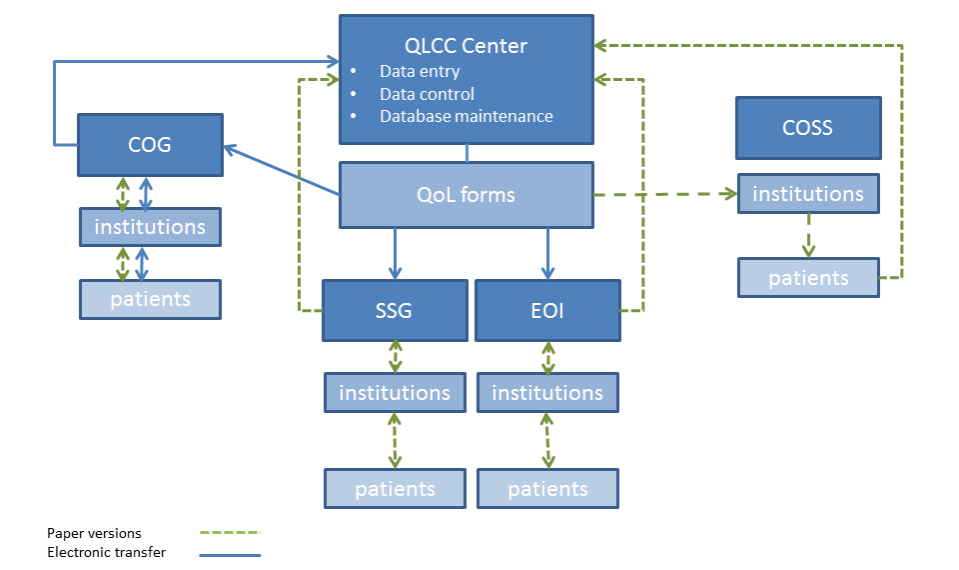
Logistics of health-related quality of life (HRQoL) assessment during EURopean AMerican Osteosarcoma Study-1 (EURAMOS-1). COG: Children’s Oncology Group; COSS: Cooperative Osteosarcoma Study; EOI: European Osteosarcoma Intergroup; QLCC: Quality of Life Coordinating Center; QoL: Quality of Life; SSG: Scandinavian Sarcoma Group.

The 4 study groups (COG, COSS, EOI, and SSG) established an infrastructure to ensure successful implementation of the EURAMOS-1 trial [19]. Ethical approval for the QoL substudy was obtained in 2005 from the ethical authority of the University Düsseldorf and subsequently in all of the participating study groups. Common data elements were agreed to standardize data collection [19]. The Quality of Life Coordinating Center (QLCC) in Germany was responsible for the QoL data storage and management. German patients returned questionnaires directly to QLCC. For other patients, the institutions sent the completed questionnaires to the national study groups, which transferred them to QLCC by post (SSG and EOI) or electronically (COG; Figure 2).

Table 1 shows the questionnaires by age and source of report. Patients’ and parents’ data were assessed independently and in parallel. QLCC was responsible for data entry, quality control, data base maintenance, and periodic reporting of the status of the QoL substudy. A comprehensive system of error checking was used to detect out-of-range or inconsistent values. If available, errors were compared with any paper records to determine the correct data values if discrepancies were found. The study was open from 2005 to 2011.

**Table 1:** Quality-of-life questionnaires according to age range and source of report.

Age range and source of report	Quality-of-life questionnaires					
	Pediatric Quality of Life Questionnaire (Calaminus et al [21])		Pediatric Quality of Life Inventory (Varni et al [24])			European Organization for Research and Treatment of Cancer—Quality of Life-Core Questionnaire C30 (Aaronson et al [20])
			
Age range (years)	≥5-7	≥8-17	≥5-7	≥8-12	>12-17	≥16
Self-reporting	+^a^	+	+	+	+	+
Proxy reporting	+	+	+	+	+	—^b^

^a^Questionnaire used.

^b^Questionnaire not used.

### Statistical Analysis

Descriptive analyses were performed for baseline patients’ characteristics as well as for the proxy reports. Categorical variables are reported as absolute and relative frequencies. Continuous variables are shown as mean, SD (±), median and range (minimum-maximum). Inferential statistical analyses were performed using Fisher exact tests for categorical variables and nonparametric methods (ie, Mann-Whitney U tests and Kruskal-Wallis tests) for continuous variables.

The comparison between QoL substudy participants and nonparticipants included a multivariable analysis using a logistic regression for modeling the probability for being a *QoL Participant*. The following variables were included in the full model: study (COG [reference category], COSS, EOI, and SSG), age (in years), gender (female vs male [reference category]), lung metastases (no and yes [reference category]), other (nonlung) metastases (no and yes [reference category]), and pathological fracture (no and yes [reference category]). COSS, EOI, and SSG also allowed participants to be defined as having possible metastases (in addition to yes and no), COG did not do so; therefore, all *possible metastases* were classified as *no metastases*.

In addition, the logistic regression was calculated for each study group separately. Odds ratios with 95% CI and Wald test *P* values were reported from the full model. Statistical analyses were performed using SAS software, version 9.4 for Windows (SAS Institute). All *P* values and CIs are exploratory without adjustment for multiplicity.

## Results

### Study Participants at Timepoint E1

**Figure 3:**
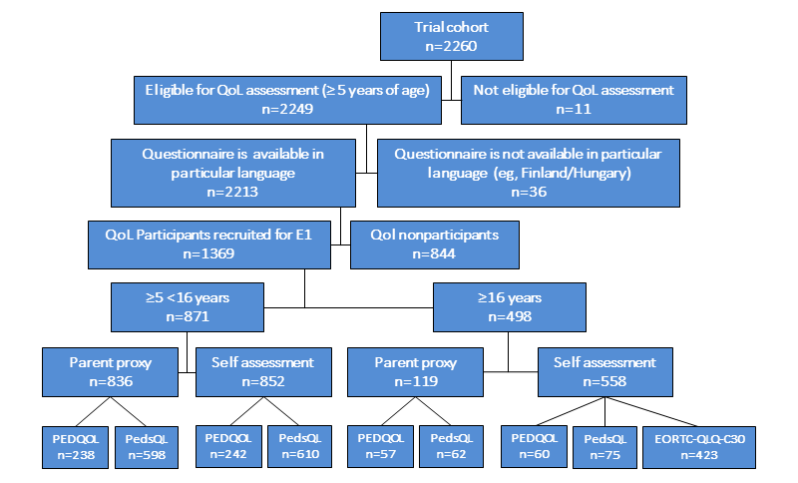
Flowchart regarding quality of life (QoL) eligibility and participation at timepoint E1, split by age and self versus proxy assessment. EORTC-QLQ-C30: European Organization for Research and Treatment of Cancer—Quality of Life-Core Questionnaire C30; PEDQOL: Pediatric Quality of Life; PedsQL: The Pediatric Quality of Life Inventory; E1: first assessment timepoint.

The EURAMOS-1 protocol registered 2260 patients who were recruited between April 2005 and June 2011. Among them, 97.92% (2213/2260) were eligible for QoL assessment and could participate in terms of questionnaire availability. For 61.86% (1369/2213) of patients, a QoL evaluation at timepoint E1 before surgery (Figure 3) was available. Nearly one-third (36.38%) of the QoL substudy participants at E1 were aged older than 16 years at timepoint E1 (n=498/1369) and 803/1369 (58.66%) were male. For the pediatric QoL substudy participants aged younger than 16 years (n=871), a completed pediatric self-assessment was available for 852 participants and a completed pediatric parent-proxy assessment was available for 836 participants (for an overview, see Figure 3). In addition, 135 patients older than 16 years filled in a pediatric questionnaire, and so finally, 987 participants with an available pediatric self-assessed questionnaire remained. Of the 987 patients with an available pediatric questionnaire at E1, 302 filled in the PEDQoL version and 685 filled in the PedsQL version (Table 2).

A similar proportion can be found for the parent-proxy assessment. In addition, 423 patients completed the questionnaire for adults (EORTC-QLQ-C30; Table 2).

As patients between 16 and 18 years of age were asked to complete both a pediatric self-assessment and an adult questionnaire according to the compilation of questionnaires agreed before the start of the study, 2 different self-assessment questionnaires are evaluable for one participant. Subsequently, the numbers of questionnaires are higher than the number of participants on the lower part of Figure 3.

Tables 2 and 3 give an overview of patient characteristics, grouped by the availability of self- and proxy reports at timepoint E1. For some patients, a proxy and a self-report are available, for others, only a proxy or a self-report is available; therefore, the numbers on proxy questionnaires are reported separately.

**Table 2:** Number and characteristics of quality-of-life substudy participants (self-reported) at timepoint 1 (E1) by questionnaire.

Characteristics			Quality-of-life questionnaires		
			Pediatric questionnaires (n=987)		Adult questionnaire (n=423)
			Pediatric Quality of Life Questionnaire (n=302)	Pediatric Quality of Life Inventory (n=685)	European Organization for Research and Treatment of Cancer—Quality of Life-Core Questionnaire C30
Sex (male), n (%)			152 (50.3)	395 (57.7)	292 (69.0)
**Age (years)**					
	≥5-<16, n (%)		242 (80.1)	610 (89.1)	—^a^
	≥**16, n (%)**		60 (19.9)	75 (10.9)	—^b^
		16-17	—^b^	—^b^	210 (49.7)
		≥18	—^a^	—^a^	213 (50.4)
	Overall mean (SD)		13.4 (2.9)	12.8 (2.9)	20.3 (5.2)
**Study groups, n (%)**					
	Children’s Oncology Group		0 (0.0)	578 (84.4)	216 (51.0)
	Cooperative Osteosarcoma Study Group		182 (60.3)	0 (0.0)	90 (21.3)
	European Osteosarcoma Intergroup		56 (18.5)	107 (15.6)	90 (21.3)
	Scandinavian Sarcoma Group		64 (21.2)	0 (0.0)	27 (6.4)
**Location site, n (%)**					
	Missing		0 (0.0)	3 (0.4)	4 (1.0)
	Upper extremity		30 (9.9)	95 (13.9)	65 (15.4)
	Lower extremity		270 (89.4)	584 (85.3)	344 (81.3)
	Other		2 (0.7)	3 (0.4)	10 (2.4)
**Lung metastases, n (%)**					
	Missing		0 (0.0)	3 (0.4)	3 (0.7)
	Yes		32 (10.6)	99 (14.5)	43 (10.2)
	No^c^		270 (89.4)	583 (85.1)	377 (89.1)
**Other (nonlung) metastases, n (%)**					
	Missing		0 (0)	3 (0.4)	3 (0.7)
	Yes		12 (4.0)	18 (2.6)	8 (1.9)
	No^c^		290 (96.0)	664 (96.9)	412 (97.4)
**Pathological fracture at diagnosis, n (%)**					
	Missing		0 (0)	4 (0.6)	3 (0.7)
	No		283 (93.7)	589 (86.0)	375 (88.7)
	Yes		19 (6.3)	92 (14.4)	45 (10.6)

^a^Questionnaire not intended for this age group.

^b^Information of this age-category is reported elsewhere in the same table.

^c^*Possible metastases* were combined with *no metastases*.

**Table 3:** Number and characteristics of quality-of-life substudy participants (proxy report) at registration by questionnaire.

Characteristics		Quality-of-life questionnaires	
		Pediatric Quality of Life Questionnaire (n=295)	Pediatric Quality of Life Inventory (n=660)
Sex (male), n (%)		147 (49.8)	388 (58.8)
**Age (years)**			
	≥5-<16, n (%)	238 (80.7)	598 (90.6)
	≥16, n (%)	57 (19.3)	62 (9.4)
	Overall mean (SD)	13.0 (2.8)	12.2 (2.9)
**Study groups, n (%)**			
	Children’s Oncology Group	N/A^a^	548 (83.0)
	Cooperative Osteosarcoma Study group	180 (61.0)	N/A
	European Osteosarcoma Intergroup	55 (18.6)	112 (17.0)
	Scandinavian Sarcoma Group	60 (20.3)	0 (0.0)

^a^N/A: not applicable.

### Participation by Timepoint

Table 4 shows how many patients provided the HRQoL self-report questionnaire, broken down by completed timepoint and all of their combinations during the study. As there were 1369 participants at E1 (Figure 3) and overall there were 1338 patients for timepoint E1, there were 31 participants at timepoint E1 with a proxy report only. The table shows a decreasing linear trend of participation with trial progress, starting with n=1338/2213 (60.46%) self-reports at timepoint 1 and continuing with n=934/2213 (42.21%) at timepoint E2 and n=668/2213(30.19%) and n=450/2213 (20.33%) at timepoints 3 and 4, respectively. However, the largest decline is between timepoint E1 and E2, and accordingly, the highest number of participants with n=454/2213 (20.52%) provided an self-assessment questionnaire at E1 but did not complete a questionnaire at any further timepoint, followed by those group of participants who provided self-assessment questionnaires at all 4 timepoints (273/2213; 12.34%).

Table 5 indicates for how many patients HRQoL proxy assessments were provided, again broken down by each timepoint and all of their combinations. With regard to Table 5, it is important to note that a proxy report was mandatory for child and adolescent patients, including those aged 17 years. Given this subgroup of 1778 patients, n=950/1778 (53.4%) of all parents of these patients provided a proxy report at timepoint E1.

**Table 4:** Number of patients with available self-assessment at any possible combination of timepoints during study (N=2213).

Timepoints during study				Patients, n (%)
E1^a^	E2^b^	E3^c^	E4^d^	
Yes	Yes	Yes	Yes	273 (12.34)
Yes	Yes	Yes	No	186 (8.40)
Yes	Yes	No	Yes	57 (2.58)
Yes	Yes	No	No	260 (11.75)
Yes	No	Yes	Yes	27 (1.22)
Yes	No	Yes	No	62 (2.80)
Yes	No	No	Yes	19 (0.86)
Yes	No	No	No	454 (20.52)
No	Yes	Yes	Yes	37 (1.67)
No	Yes	Yes	No	42 (1.90)
No	Yes	No	Yes	6 (0.27)
No	Yes	No	No	73 (3.30)
No	No	Yes	Yes	11 (0.50)
No	No	Yes	No	30 (1.36)
No	No	No	Yes	20 (0.90)
No	No	No	No	656 (29.64)

^a^Timepoint 1.

^b^Timepoint 2.

^c^Timepoint 3.

^d^Timepoint 4.

**Table 5:** Number of patients with an available proxy assessment at any possible combination of timepoints during the study (N=2213).

Timepoints during study				Patients n (%)
E1^a^	E2^b^	E3^c^	E4^d^	
Yes	Yes	Yes	Yes	155 (7.00)
Yes	Yes	Yes	No	159 (7.18)
Yes	Yes	No	Yes	36 (1.63)
Yes	Yes	No	No	223 (10.08)
Yes	No	Yes	Yes	22 (0.99)
Yes	No	Yes	No	36 (1.63)
Yes	No	No	Yes	9 (0.41)
Yes	No	No	No	320 (14.46)
No	Yes	Yes	Yes	19 (0.86)
No	Yes	Yes	No	33 (1.49)
No	Yes	No	Yes	5 (0.23)
No	Yes	No	No	74 (3.34)
No	No	Yes	Yes	9 (0.41)
No	No	Yes	No	17 (0.77)
No	No	No	Yes	8 (0.36)
No	No	No	No	1088 (49.16)

^a^Timepoint 1.

^b^Timepoint 2.

^c^Timepoint 3.

^d^Timepoint 4.

### Quality of Life—Nonparticipant Analyses at Timepoint E1

The nonparticipant analyses were performed on patient level if any age-appropriate questionnaire (self- or proxy report or pediatric or adult questionnaire) was available at timepoint E1. Demographic and disease-related characteristics for QoL substudy participants and nonparticipants at timepoint E1 of the QoL study are listed in Table 6. No statistically significant differences in the participation rates with respect to age or gender were seen. Participation rates differed substantially between national study groups (67% COG, 80% SSG, 56% EOI, and 50% COSS). Patients from the COSS group and EOI were less likely to participate in the QoL study’s baseline assessment (OR 0.48, 95% CI 0.386-0.600 and OR 0.61, 95% CI 0.482-0.759, respectively) compared with patients from COG, whereas patients from the SSG had a higher participation rate than those from COG (OR 1.97, 95% CI 1.223-3.172).

**Table 6:** Comparison of quality of life (QoL) nonparticipants and QoL participants at timepoint 1 (E1) in the overall cohort (N=2213).

Characteristics		QoL overall sample at registration	
		QoL nonparticipants (n=844)	QoL participants (n=1369)
Sex (male), n (%)		498 (59.00)	803 (58.66)
**Age (years)**			
	≥5-<16, n (%)	523 (61.97)	871 (63.62)
	≥16, n (%)	321 (38.03)	498 (35.94)
	Overall mean (SD)	15.4 (5.6)	15.0 (5.10)
**Study groups, n (%)**			
	Children’s Oncology Group	380 (45.02)	784 (57.27)
	Cooperative Osteosarcoma Study group	242 (28.67)	241 (17.60)
	European Osteosarcoma Intergroup	199 (23.58)	252 (18.41)
	Scandinavian Sarcoma Group	23 (2.73)	92 (6.72)
**Tumor location, n (%)**			
	Missing	9 (1.07)	7 (0.51)
	Upper extremity	130 (15.40)	188 (13.73)
	Lower extremity	700 (82.94)	1159 (84.66)
	Other	5 (0.59)	15 (1.10)
**Lung metastases, n (%)**			
	Missing	9 (1.07)	6 (0.44)
	Yes	125 (14.81)	170 (12.42)
	No^a^	710 (84.12)	1193 (87.14)
**Other (nonlung) metastases, n (%)**			
	Missing	11 (1.30)	6 (0.44)
	Yes	35 (4.15)	37 (2.70)
	No^a^	798 (94.55)	1326 (96.86)
**Pathological fracture at diagnosis, n (%)**			
	Missing	11 (1.30)	7 (0.51)
	No	725 (85.90)	1202 (87.80)
	Yes	108 (12.80)	160 (11.69)

^a^*Possible metastases* were combined with *no metastases*.

There was no evidence that age (*P*=.27) or gender (*P*=.61) influenced participation. Individual models for each study group revealed that within the COG group, female patients were less likely to participate (OR 0.76, 95% CI 0.585-0.974; *P*=.03), whereas female patients were more likely to participate within the COSS group (OR 1.57, 95% CI 1.070-2.29; *P*=.02). For the EOI and the SSG, gender did not have an influence on the participation.

Considering participation rates at timepoint E1 with regard to disease characteristics, no differences in participation were obtained with regard to major tumor site (*P*=.29), occurrence of pathological fracture at diagnosis (*P*=.13), and initial presentation with metastasis (lung; *P*=.10), or other sites (*P*=.13) before registration. Patients with femur as the primary site contributed most frequently to the baseline QoL assessments (695; 51.0%), whereas patients with radius as the primary site participated relatively less frequently (16; 1.2%).

## Discussion

### Principal Findings

Here, we describe the QoL substudy embedded in the international OS trial, EURAMOS-1. A total of 4 national study groups that included 17 countries contributed to the study and resulted in the first prospective QoL international study of OS. We collected at least at timepoint E1 QoL information from nearly 1400 patients with an age range from 5 to 40 years. Most reported OS QoL studies were smaller and/or have focused only on posttreatment or were not prospective from the time of diagnosis [8-10]. This prospective study aimed to provide information not previously reported in the literature and results will help to develop interventions to improve QoL.

Overall, in EURAMOS-1, there were no differences in participation rates at timepoint E1 with regard to the age, gender, site of primary tumor involvement, occurrence of pathological fracture, or occurrence of lung or other metastases at diagnosis between QoL substudy participants and nonparticipants. However, a difference of participation rates between the different national study groups was evident. Logistic regression revealed that patients from the COG were more likely to participate in the QoL assessment compared with COSS and EOI patients, but they were less likely to participate compared with patients from the SSG. In addition, within national study groups (COSS and COG), some significant differences in gender participation were seen. Beside differences in the national study group structure, cultural tendencies could have influenced the different response rates. Further investigation into these differences is warranted.

### Comparison With Previous Work

Although there was a participation rate of about 60% at timepoint E1 and the participation rates regarding self-assessment decreased to just over 20% at timepoint E4, this is reasonable given the characteristics of the study cohort (eg, in terms of participant’s age range and the number of countries involved). In other HRQoL studies involving children with cancer, the response rates have varied between 58% and 98% [29]. A recent multicenter prospective study including children with lymphoblastic leukemia reported a participation rate of 63% and obtained also a less pronounced but substantial linear decline of participation with study progress [30]. One must keep in mind that investigating QoL in adolescents and young adults (AYAs) may be even more difficult (in this study, 56.5% were aged ≥13 years) because of their developmental status and pursuit of autonomy. These circumstances probably influenced the participation rates. In addition, declining participation rates over the course of the trial are also influenced by overall and event-free survival (eg, at 36 months after biopsy, they were approximately 80% and 60%, respectively) [31]. Rosenberg’s study [32] of only AYA patients reported a response rate of only 74% at a single timepoint, even when patients were rewarded for participation. There are no comparable sarcoma trials that include QoL and cover such a broad study age range from various countries and ethnicities. Compared with other international QoL substudies in adult cancer RCTs, these studies obtained higher participation rates at baseline in different diagnoses, for example, leukemia [33] or ovarian cancer [34,35]. However, these trials differed also in terms of assessment method. For instance, provision of an electronic device may have increased the likelihood of participating in the study by Topp et al [33]. Moreover, only adult but no adolescent patients were included. In the future, this could be improved by incorporating Web-based or mobile apps, according to the recommendation by Johnston et al [29] who explored reasons for nonparticipation in QoL studies on children with cancer and their parents. They came to the conclusion that this may address many logistical challenges.

### Limitations

Some limitations have to be addressed. We did not assess the level of baseline pain or the extent of anxiety regarding the diagnosis or the urgency to start treatment; both variables may have an important influence on participation rate. Large sample sizes increase the chance of obtaining significant results. Therefore, our results have to be interpreted cautiously. The unavailability of a common QoL questionnaire usable across all groups/countries is a limitation in comparison across groups but was unavoidable. Given the large number of participating sites, how the questionnaires were provided to families and administered may lead to differences. This prospective design does allow analysis of changes over time and at an intraindividual level; however, the representativeness of the collected data may be affected by low participation rates (particularly in respect to the reduced sample size at timepoint E4). In addition, the comparability of measurements is limited as the questionnaires changed (at the age of 16 years) between timepoints and differed between national study groups; participants could also choose to participate only at certain timepoints. To overcome this obstacle, we plan on using a linking method for score conversion [36] for the subscales with sufficient conceptual overlap between questionnaires. We will base the linking of scores on a subset of participants who completed 2 questionnaires at the same timepoint (*single-group design*) according to schedule. We will assess concordance using appropriate measures (eg, Bland-Altman plots and Lin correlation concordance coefficient) [37,38].

### Conclusions

Despite some of the limitations, this is largest prospective assessment of QoL in OS therapy. Further analyses will be able to look at prospective changes and be able to look at long-term outcomes and differences between different demographic groups. This study also highlights the ability of clinicians and researchers to work together to perform large QoL investigations across different national study groups. For such an endeavor to succeed, there needed to support from each national study group that includes first recognizing the importance of QoL assessments, the provision of infrastructure for the collection and management of QoL data, the identification of *lead* QoL investigators for each group, and time/support for meeting as a group. Through the initial phases of the development of this study, we needed to come together to identify the most appropriate QoL questionnaires that were available at the time that it was validated for the different involved countries/languages, had similar domains, and can span the age range of participants. We then had to determine how often and when assessments will be done while accounting for structural differences in the various groups/countries in how they delivered therapy and administered questionnaires and ensuring that questionnaires were not too burdensome. After intensive discussions, compromises were made regarding the timing of initial QoL assessment and the number of assessments. In terms of administration, it was decided that the administration and tracking of the assessments has to be in the hands of the national study groups and that centralized administration would not be possible. However, the data management and quality control checks were centralized with the QLCC taking the lead. Each national study group determined its own system of transferring the QoL forms/data to the QLCC. Overall, this was a successful endeavor, and we hope learnings from this partnership will lead to future studies. With this study, future analyses will lead to a better understanding of the impact OS therapy has on QoL and how patient and particular disease characteristics influence QoL in the short and long term and how QoL changes over time. This will help to ameliorate or prevent the potential decline in psychosocial and physical morbidities of patients undergoing OS treatment.

## Appendix

Multimedia Appendix 1 Participating countries listed by study group.

## Abbreviations

AYA: adolescents and young adults
COG: Children’s Oncology Group
COSS: Cooperative Osteosarcoma Study
EOI: European Osteosarcoma Intergroup
EORTC-QLQ-C30: European Organization for Research and Treatment of Cancer—Quality of Life-Core Questionnaire C30
EURAMOS-1: EURopean AMerican Osteosarcoma Study-1
HRQoL: health-related quality of life
NCTN: National Clinical Trial Network
OS: osteosarcoma
QLCC: Quality of Life Coordinating Center
QoL: quality of life
SSG: Scandinavian Sarcoma Group

## References

[1] Bielack SS, Kempf-Bielack B, Delling G, Exner GU, Flege S, Helmke K, Kotz R, Salzer-Kuntschik M, Werner M, Winkelmann W, Zoubek A, Jürgens H, Winkler K (2002). Prognostic factors in high-grade osteosarcoma of the extremities or trunk: an analysis of 1,702 patients treated on neoadjuvant cooperative osteosarcoma study group protocols. J Clin Oncol.

[2] Esiashvili N, Goodman M, Marcus Jr RB (2008). Changes in incidence and survival of Ewing sarcoma patients over the past 3 decades: surveillance epidemiology and end results data. J Pediatr Hematol Oncol.

[3] Ferrari S, Pieretti F, Verri E, Tolentinis L, Cesari M, Versari M, Zolezzi C, Lamanna G, Bacci G (2005). Prospective evaluation of renal function in pediatric and adult patients treated with high-dose ifosfamide, cisplatin and high-dose methotrexate. Anticancer Drugs.

[4] Lewis IJ, Nooij MA, Whelan J, Sydes MR, Grimer R, Hogendoorn PC, Memon MA, Weeden S, Uscinska BM, van Glabbeke M, Kirkpatrick A, Hauben EI, Craft AW, Taminiau AH, MRC BO06EORTC 80931 Collaborators, European Osteosarcoma Intergroup (2007). Improvement in histologic response but not survival in osteosarcoma patients treated with intensified chemotherapy: a randomized phase III trial of the European Osteosarcoma Intergroup. J Natl Cancer Inst.

[5] Meyers PA, Schwartz CL, Krailo M, Kleinerman ES, Betcher D, Bernstein ML, Conrad E, Ferguson W, Gebhardt M, Goorin AM, Harris MB, Healey J, Huvos A, Link M, Montebello J, Nadel H, Nieder M, Sato J, Siegal G, Weiner M, Wells R, Wold L, Womer R, Grier H (2005). Osteosarcoma: a randomized, prospective trial of the addition of ifosfamide and/or muramyl tripeptide to cisplatin, doxorubicin, and high-dose methotrexate. J Clin Oncol.

[6] Mirabello L, Troisi RJ, Savage SA (2009). Osteosarcoma incidence and survival rates from 1973 to 2004: data from the surveillance, epidemiology, and end results program. Cancer.

[7] Meyers PA, Schwartz CL, Krailo MD, Healey JH, Bernstein ML, Betcher D, Ferguson WS, Gebhardt MC, Goorin AM, Harris M, Kleinerman E, Link MP, Nadel H, Nieder M, Siegal GP, Weiner MA, Wells RJ, Womer RB, Grier HE, Children's Oncology Group (2008). Osteosarcoma: the addition of muramyl tripeptide to chemotherapy improves overall survival--a report from the Children's Oncology Group. J Clin Oncol.

[8] van Riel CA, Meijer-van den Bergh EE, Kemps HL, Feuth T, Schreuder HW, Hoogerbrugge PM, de Groot IJ, Mavinkurve-Groothuis AM (2014). Self-perception and quality of life in adolescents during treatment for a primary malignant bone tumour. Eur J Oncol Nurs.

[9] Bekkering WP, Vlieland TP, Koopman HM, Schaap GR, Schreuder HW, Beishuizen A, Tissing WJ, Hoogerbrugge PM, Anninga JK, Taminiau AH (2010). Quality of life in young patients after bone tumor surgery around the knee joint and comparison with healthy controls. Pediatr Blood Cancer.

[10] Eiser C, Darlington AS, Stride CB, Grimer R (2001). Quality of life implications as a consequence of surgery: limb salvage, primary and secondary amputation. Sarcoma.

[11] Stokke J, Sung L, Gupta A, Lindberg A, Rosenberg AR (2015). Systematic review and meta-analysis of objective and subjective quality of life among pediatric, adolescent, and young adult bone tumor survivors. Pediatr Blood Cancer.

[12] Nagarajan R, Neglia JP, Clohisy DR, Robison LL (2002). Limb salvage and amputation in survivors of pediatric lower-extremity bone tumors: what are the long-term implications?. J Clin Oncol.

[13] Bekkering WP, Vlieland TP, Koopman HM, Schaap GR, Beishuizen A, Anninga JK, Wolterbeek R, Nelissen RG, Taminiau AH (2012). A prospective study on quality of life and functional outcome in children and adolescents after malignant bone tumor surgery. Pediatr Blood Cancer.

[14] Hinds PS, Billups CA, Cao X, Gattuso JS, Burghen E, West N, Rubnitz JE, Daw NC (2009). Health-related quality of life in adolescents at the time of diagnosis with osteosarcoma or acute myeloid leukemia. Eur J Oncol Nurs.

[15] Hinds PS, Gattuso JS, Billups CA, West NK, Wu J, Rivera C, Quintana J, Villarroel M, Daw NC (2009). Aggressive treatment of non-metastatic osteosarcoma improves health-related quality of life in children and adolescents. Eur J Cancer.

[16] Bielack SS, Smeland S, Whelan JS, Marina N, Jovic G, Hook JM, Krailo MD, Gebhardt M, Pápai Z, Meyer J, Nadel H, Randall RL, Deffenbaugh C, Nagarajan R, Brennan B, Letson GD, Teot LA, Goorin A, Baumhoer D, Kager L, Werner M, Lau CC, Sundby HK, Gelderblom H, Meyers P, Gorlick R, Windhager R, Helmke K, Eriksson M, Hoogerbrugge PM, Schomberg P, Tunn P, Kühne T, Jürgens H, van den Berg H, Böhling T, Picton S, Renard M, Reichardt P, Gerss J, Butterfass-Bahloul T, Morris C, Hogendoorn PC, Seddon B, Calaminus G, Michelagnoli M, Dhooge C, Sydes MR, Bernstein M, EURAMOS-1 Investigators (2015). Methotrexate, Doxorubicin, and Cisplatin (MAP) plus maintenance Pegylated Interferon Alfa-2b versus MAP alone in patients with resectable high-grade osteosarcoma and good histologic response to preoperative MAP: first results of the EURAMOS-1 good response randomized controlled trial. J Clin Oncol.

[17] Marina NM, Smeland S, Bielack SS, Bernstein M, Jovic G, Krailo MD, Hook JM, Arndt C, van den Berg H, Brennan B, Brichard B, Brown KL, Butterfass-Bahloul T, Calaminus G, Daldrup-Link HE, Eriksson M, Gebhardt MC, Gelderblom H, Gerss J, Goldsby R, Goorin A, Gorlick R, Grier HE, Hale JP, Hall KS, Hardes J, Hawkins DS, Helmke K, Hogendoorn PC, Isakoff MS, Janeway KA, Jürgens H, Kager L, Kühne T, Lau CC, Leavey PJ, Lessnick SL, Mascarenhas L, Meyers PA, Mottl H, Nathrath M, Papai Z, Randall RL, Reichardt P, Renard M, Safwat AA, Schwartz CL, Stevens MC, Strauss SJ, Teot L, Werner M, Sydes MR, Whelan JS (2016). Comparison of MAPIE versus MAP in patients with a poor response to preoperative chemotherapy for newly diagnosed high-grade osteosarcoma (EURAMOS-1): an open-label, international, randomised controlled trial. Lancet Oncol.

[18] Whelan JS, Bielack SS, Marina N, Smeland S, Jovic G, Hook JM, Krailo M, Anninga J, Butterfass-Bahloul T, Böhling T, Calaminus G, Capra M, Deffenbaugh C, Dhooge C, Eriksson M, Flanagan AM, Gelderblom H, Goorin A, Gorlick R, Gosheger G, Grimer RJ, Hall KS, Helmke K, Hogendoorn PC, Jundt G, Kager L, Kuehne T, Lau CC, Letson GD, Meyer J, Meyers PA, Morris C, Mottl H, Nadel H, Nagarajan R, Randall RL, Schomberg P, Schwarz R, Teot LA, Sydes MR, Bernstein M, EURAMOS Collaborators (2015). EURAMOS-1, an international randomised study for osteosarcoma: results from pre-randomisation treatment. Ann Oncol.

[19] Marina NM, Bielack S, Whelan J, Smeland S, Krailo M, Sydes MR, Butterfass-Bahloul T, Calaminus G, Bernstein M (2009). International collaboration is feasible in trials for rare conditions: the EURAMOS experience. Cancer Treat Res.

[20] Aaronson NK, Ahmedzai S, Bergman B, Bullinger M, Cull A, Duez NJ, Filiberti A, Flechtner H, Fleishman SB, de Haes JC (1993). The European Organization for Research and Treatment of Cancer QLQ-C30: a quality-of-life instrument for use in international clinical trials in oncology. J Natl Cancer Inst.

[21] Calaminus G, Weinspach S, Teske C, Göbel U (2000). Quality of life in children and adolescents with cancer. First results of an evaluation of 49 patients with the PEDQOL questionnaire. Klin Padiatr.

[22] Calaminus G, Weinspach S, Teske C, Göbel U (2007). Quality of survival in children and adolescents after treatment for childhood cancer: the influence of reported late effects on health related quality of life. Klin Padiatr.

[23] Varni JW, Seid M, Kurtin PS (2001). PedsQL 4.0: reliability and validity of the Pediatric Quality of Life Inventory version 4.0 generic core scales in healthy and patient populations. Med Care.

[24] Varni JW, Burwinkle TM, Katz ER, Meeske K, Dickinson P (2002). The PedsQL in pediatric cancer: reliability and validity of the Pediatric Quality of Life Inventory Generic Core Scales, Multidimensional Fatigue Scale, and Cancer Module. Cancer.

[25] Heutte N, Flechtner HH, Mounier N, Mellink WA, Meerwaldt JH, Eghbali H, van't Veer MB, Noordijk EM, Kluin-Nelemans JC, Lampka E, Thomas J, Lugtenburg PJ, Viterbo L, Carde P, Hagenbeek A, van der Maazen RW, Smit WG, Brice P, van Marwijk KM, Baars JW, Poortmans P, Tirelli U, Leeksma OC, Tomsic R, Feugier P, Salles G, Gabarre J, Kersten MJ, van den Neste E, Creemers GM, Gaillard I, Meijnders P, Tertian G, Reman O, Muller HP, Troncy J, Blanc M, Schroyens W, Voogt PJ, Wijermans P, Rieux C, Fermé C, Henry-Amar M, EORTC-GELA H8 Trial Group (2009). Quality of life after successful treatment of early-stage Hodgkin's lymphoma: 10-year follow-up of the EORTC-GELA H8 randomised controlled trial. Lancet Oncol.

[26] Schwarz R, Hinz A (2001). Reference data for the quality of life questionnaire EORTC QLQ-C30 in the general German population. Eur J Cancer.

[27] Upton P, Eiser C, Cheung I, Hutchings HA, Jenney M, Maddocks A, Russell IT, Williams JG (2005). Measurement properties of the UK-english version of the Pediatric Quality of Life Inventory 4.0 (PedsQL) generic core scales. Health Qual Life Outcomes.

[28] Varni JW, Burwinkle TM, Seid M (2006). The PedsQL 4.0 as a school population health measure: feasibility, reliability, and validity. Qual Life Res.

[29] Johnston DL, Nagarajan R, Caparas M, Schulte F, Cullen P, Aplenc R, Sung L (2013). Reasons for non-completion of health related quality of life evaluations in pediatric acute myeloid leukemia: a report from the Children's Oncology Group. PLoS One.

[30] Eiser C, Stride CB, Vora A, Goulden N, Mitchell C, Buck G, Adams M, Jenney ME, National Cancer Research Institute Childhood Leukaemia Sub-Group and UK Childhood Leukaemia Clinicians Network (2017). Prospective evaluation of quality of life in children treated in UKALL 2003 for acute lymphoblastic leukaemia: a cohort study. Pediatr Blood Cancer.

[31] Smeland S, Bielack SS, Whelan J, Bernstein M, Hogendoorn P, Krailo MD, Gorlick R, Janeway KA, Ingleby FC, Anninga J, Antal I, Arndt C, Brown KL, Butterfass-Bahloul T, Calaminus G, Capra M, Dhooge C, Eriksson M, Flanagan AM, Friedel G, Gebhardt MC, Gelderblom H, Goldsby R, Grier HE, Grimer R, Hawkins DS, Hecker-Nolting S, Sundby HK, Isakoff MS, Jovic G, Kühne T, Kager L, von Kalle T, Kabickova E, Lang S, Lau CC, Leavey PJ, Lessnick SL, Mascarenhas L, Mayer-Steinacker R, Meyers PA, Nagarajan R, Randall RL, Reichardt P, Renard M, Rechnitzer C, Schwartz CL, Strauss S, Teot L, Timmermann B, Sydes MR, Marina N (2019). Survival and prognosis with osteosarcoma: outcomes in more than 2000 patients in the EURAMOS-1 (European and American osteosarcoma study) cohort. Eur J Cancer.

[32] Rosenberg AR, Bona K, Wharton CM, Bradford M, Shaffer ML, Wolfe J, Baker KS (2016). Adolescent and young adult patient engagement and participation in survey-based research: a report from the 'resilience in adolescents and young adults with cancer' study. Pediatr Blood Cancer.

[33] Topp MS, Zimmerman Z, Cannell P, Dombret H, Maertens J, Stein A, Franklin J, Tran Q, Cong Z, Schuh AC (2018). Health-related quality of life in adults with relapsed/refractory acute lymphoblastic leukemia treated with blinatumomab. Blood.

[34] Vergote IB, Jimeno A, Joly F, Katsaros D, Coens C, Despierre E, Marth C, Hall M, Steer CB, Colombo N, Lesoin A, Casado A, Reinthaller A, Green J, Buck M, Ray-Coquard I, Ferrero A, Favier L, Reed NS, Curé H, Pujade-Lauraine E (2014). Randomized phase III study of erlotinib versus observation in patients with no evidence of disease progression after first-line platin-based chemotherapy for ovarian carcinoma: a European organisation for research and treatment of cancer-gynaecological cancer group, and gynecologic cancer intergroup study. J Clin Oncol.

[35] Stark D, Nankivell M, Pujade-Lauraine E, Kristensen G, Elit L, Stockler M, Hilpert F, Cervantes A, Brown J, Lanceley A, Velikova G, Sabate E, Pfisterer J, Carey MS, Beale P, Qian W, Swart AM, Oza A, Perren T (2013). Standard chemotherapy with or without bevacizumab in advanced ovarian cancer: quality-of-life outcomes from the International Collaboration on Ovarian Neoplasms (ICON7) phase 3 randomised trial. Lancet Oncol.

[36] Kolen MJ, Brennan RL (2014). Test Equating, Scaling, and Linking: Methods and Practices. Third Edition.

[37] Lin LI (1989). A concordance correlation coefficient to evaluate reproducibility. Biometrics.

[38] Bland JM, Altman DG (1986). Statistical methods for assessing agreement between two methods of clinical measurement. Lancet.

