# Supplementary material for: Quality of Life of Patients With Osteosarcoma in the European American Osteosarcoma Study-1 (EURAMOS-1): Development and Implementation of a Questionnaire Substudy
Source: JMIR Res Protoc. 2019 Sep 26;8(9):e14406. doi: 10.2196/14406 (PMC6819132; doi:10.2196/14406)
Supplement: Multimedia Appendix 1 [file resprot_v8i9e14406_app1.pdf]

Multimedia Appendix 1

Participating countries listed by study group

Children's Oncology Group (COG)

Australia
Canada
New Zealand
Parts of Switzerland
USA

Cooperative Osteosarcoma Study group (COSS)

Austria
Czech Republic
Germany
Hungary
Parts of Switzerland

European Osteosarcoma Intergroup (EOI)

Belgium
Republic of Ireland
The Netherlands
United Kingdom

Scandinavian Sarcoma Group (SSG)

Denmark
Finland
Norway
Sweden
